# Supplementary material for: Mechanisms of Transmission Ratio Distortion at Hybrid Sterility Loci Within and Between Mimulus Species
Source: G3 (Bethesda). 2017 Sep 20;7(11):3719–30. doi: 10.1534/g3.117.300148 (PMC5677164; doi:10.1534/g3.117.300148)
Supplement: Supplementary file 1 [file 3719FigureS1.pdf]

Figure S1

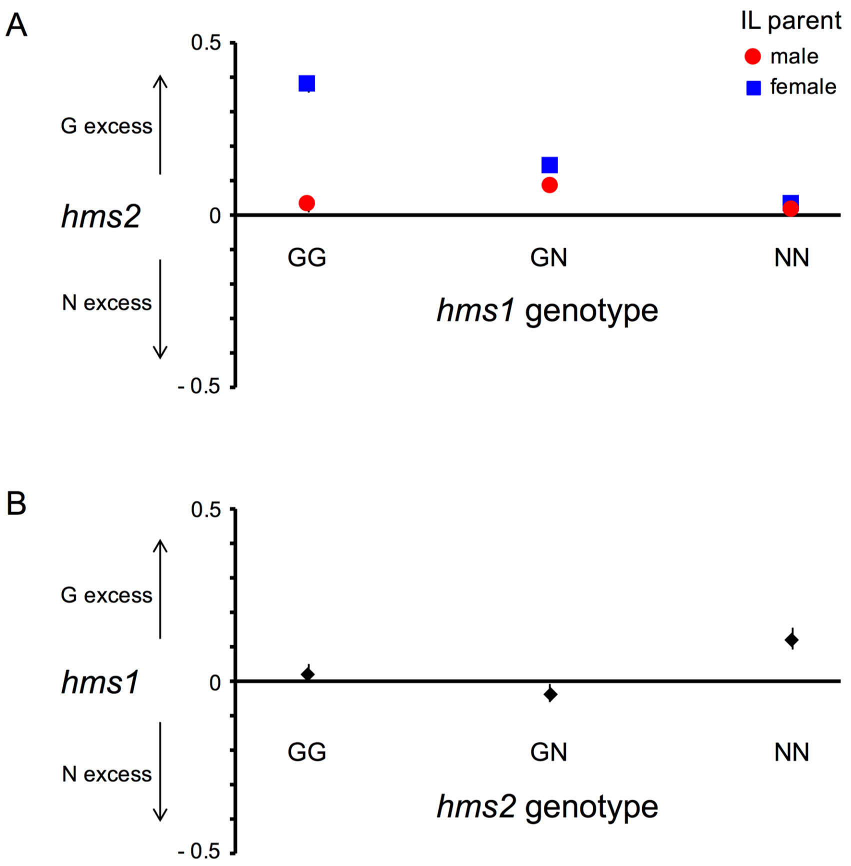

Figure S1. Transmission ratio distortion at *hms1* and *hms2* in IL-backcross progeny. The vertical position of each symbol shows the magnitude and direction of the deviation of allelic transmission from the Mendelian expectation (0.5). *M. guttatus* deviations are graphed directly [deviation = f(N – 0.5)], and the *M. nasutus* deviations are graphed as negative [deviation = - (f(N-0.25)]. Thus, values above zero indicate excess of *M. guttatus* alleles and values below zero indicate excess of *M. nasutus* alleles. G = *M. guttatus* allele, N = *M. nasutus* allele. A) Allelic transmission of *hms2* in the progeny of IL-backcrosses is significantly affected by IL parental genotype at *hms1* (F = 37.6919, P < 0.0001), cross direction (F = 72.3339, P < 0.0001), and their interaction (F = 31.8353, P < 0.0001). B) Allelic transmission of *hms1* in the progeny of IL-backcrosses is significantly affected by IL parental genotype at *hms1* (F = 7.7977, P = 0.0043).
