# Supplementary material for: Mechanisms of Transmission Ratio Distortion at Hybrid Sterility Loci Within and Between Mimulus Species
Source: G3 (Bethesda). 2017 Sep 20;7(11):3719–30. doi: 10.1534/g3.117.300148 (PMC5677164; doi:10.1534/g3.117.300148)
Supplement: Supplementary file 2 [file 3719FigureS2.pdf]

Figure S2

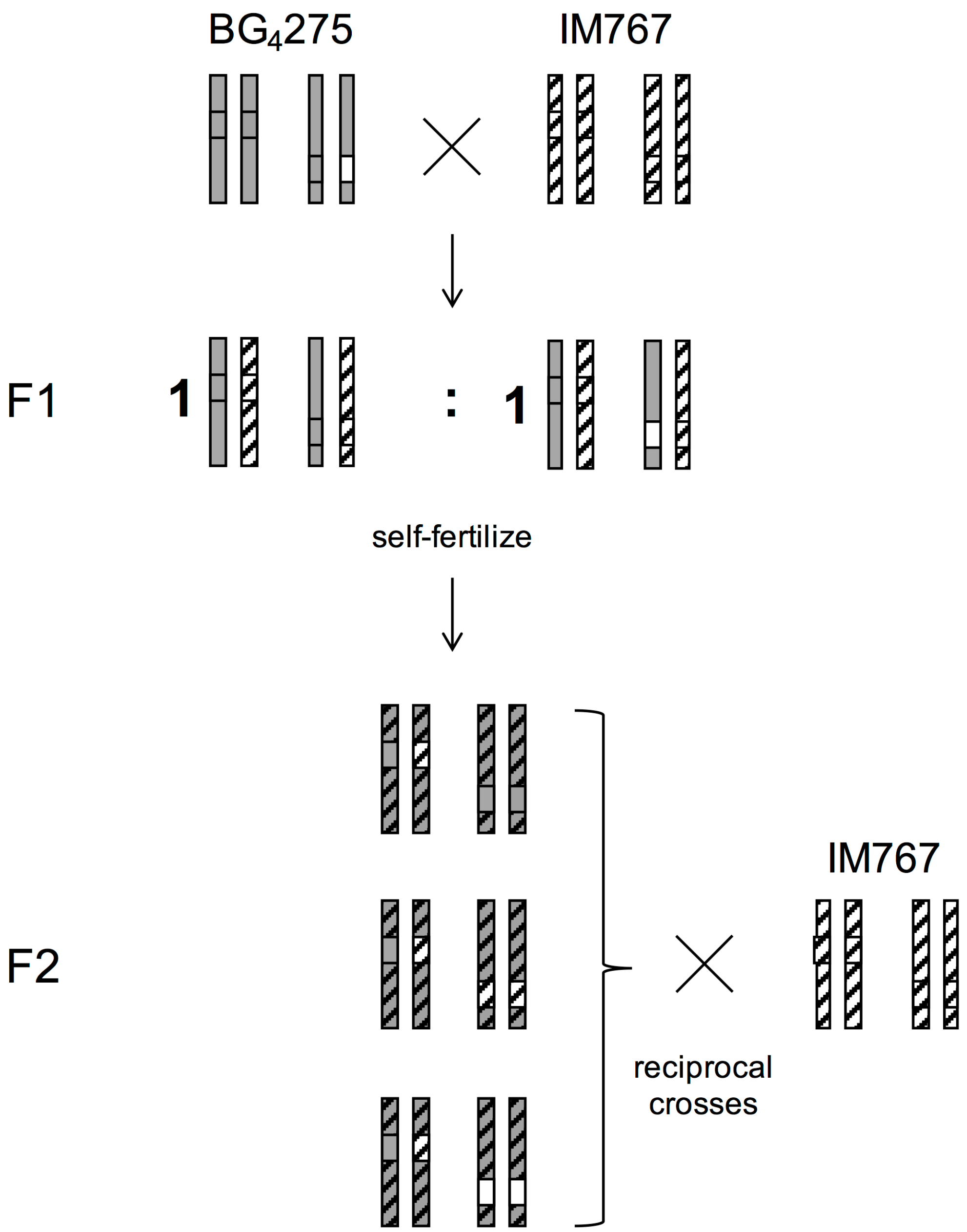

Figure S2. Crossing scheme for investigating the effect of *M. nasutus* (SF5) homozygosity at *hms2* on within-*M. guttatus* TRD at *hms1*. For each genotype, two chromosome pairs are shown (one with *hms1* and one with *hms2*). We intercrossed IM767 (stripes) and BG$_4$.275, which carries a heterozygous SF5 introgression (white) at *hms2* against an IM62 (grey shading) genetic background. The resulting progeny segregate 1:1 for two different genotypes at *hms2*: IM62-IM767 heterozygotes and SF5-IM767 heterozygotes (the remaining genetic background is heterozygous for IM62 and IM767 alleles). We genotyped F$_2$ progeny with *hms*-linked markers to identify IM62-IM767 *hms1* heterozygotes in combination with three different *hms2* genotypes: IM62 homozygotes, IM767 homozygotes, and SF5 homozygotes. Individuals with each of these three two-locus genotypes were then reciprocally backcrossed to IM767 to assess TRD at *hms1*. Note that the genetic background of the F$_2$ progeny are expected to segregate 1:2:1 for IM62 homozygotes, heterozygotes, and IM767 homozygotes (grey shading with stripes).
