# Supplementary material for: Mechanisms of Transmission Ratio Distortion at Hybrid Sterility Loci Within and Between Mimulus Species
Source: G3 (Bethesda). 2017 Sep 20;7(11):3719–30. doi: 10.1534/g3.117.300148 (PMC5677164; doi:10.1534/g3.117.300148)
Supplement: Supplementary file 3 [file 3719TableS1.docx]

Table S1. Observed and expected genotype frequencies at *hms1* and *hms2* in F_2_ hybrids (*N* = 5487).

| genotype  *hms1*; *hms2* | O | E: Mendelian | E: GN inviable, 1 parent^1^ | E: GN inviable, 2 parents^2^ | E: GN partial inviablity, 1 parent^3^ |
| --- | --- | --- | --- | --- | --- |
| GG; GG | 0.099 | 0.0625 | 0.083 | 0.109 | 0.076 |
| GG; GN | 0.100 | 0.1250 | 0.083 | 0 | 0.098 |
| GG; NN | 0.022 | 0.0625 | 0 | 0 | 0.022 |
| GN; GG | 0.208 | 0.1250 | 0.165 | 0.218 | 0.152 |
| GN; GN | 0.268 | 0.2500 | 0.248 | 0.218 | 0.25 |
| GN; NN | 0.071 | 0.1250 | 0.083 | 0 | 0.098 |
| NN; GG | 0.070 | 0.0625 | 0.083 | 0.109 | 0.076 |
| NN; GN | 0.117 | 0.1250 | 0.165 | 0.218 | 0.152 |
| NN; NN | 0.047 | 0.0625 | 0.083 | 0.109 | 0.076 |

^1^ Expected F_2_ genotype frequencies if 100% of *hms1*_G_; *hms2*_N_ gametes are inviable in one parent. Observed F_2_ genotype counts significantly differ from this expectation (*X* ^2^ = 325.725, d.f. = 8, *P* <0.0001). G = *M. guttatus* allele, N = *M. nasutus* allele.

^2^ Expected F_2_ genotype frequencies if 100% of *hms1*_G_; *hms2*_N_ gametes are inviable in both parents. Observed F_2_ genotype counts significantly differ from this expectation (*X* ^2^ = 1853.55, d.f. = 8, *P* <0.0001).

^3^ Expected F_2_ genotype frequencies if 65% of *hms1*_G_; *hms2*_N_ gametes are inviable in one parent. This threshold of inviability was set by assuming the observed *hms1*_GG_; *hms2*_NN_ F_2_ genotype frequency was determined solely by partial inviability through one parent. Observed F_2_ genotype counts significantly differ from this expectation (*X* ^2^ = 156.892, d.f. = 8, *P* <0.0001).
