# Supplementary material for: Mechanisms of Transmission Ratio Distortion at Hybrid Sterility Loci Within and Between Mimulus Species
Source: G3 (Bethesda). 2017 Sep 20;7(11):3719–30. doi: 10.1534/g3.117.300148 (PMC5677164; doi:10.1534/g3.117.300148)
Supplement: Supplementary file 4 [file 3719TableS2.docx]

Table S2. The severity of under-transmission of *hms1*_G_; *hms2*_N_ gametes (measured as the deviation from the Mendelian expectation of 0.25) in IL-backcrosses is affected by genetic background, cross direction, and identity of the recurrent parent.

| Effect^1^ |  | df | *F* | *P* |  | LSM | |
| --- | --- | --- | --- | --- | --- | --- | --- |
| Background^2^ |  | 1 | 8.259 | 0.045 |  | G: -0.095 | N: -0.149 |
| Cross direction^3^ |  | 1 | 30.910 | 0.005 |  | ♂: -0.174 | ♀: -0.070 |
| Recurrent parent^4^ |  | 1 | 7.359 | 0.053 |  | G: -0.147 | N: -0.097 |

^1^Effects assessed by ANOVA with degrees of freedom (df), F-ratio (*F*), p-values (*P*), and least squares means (LSM) indicated.

^2^Crosses performed using fourth-generation NILs. *M. nasutus* background = BN_4_; *M. guttatus* background = BG_4_.

^3^Cross direction indicates whether the NIL was used as the paternal (♂) or maternal (♀) parent.

^4^Testcrosses were to the IM62 line of *M. guttatus* (G) or the SF line of *M. nasutus* (N).
