# Supplementary material for: Mechanisms of Transmission Ratio Distortion at Hybrid Sterility Loci Within and Between Mimulus Species
Source: G3 (Bethesda). 2017 Sep 20;7(11):3719–30. doi: 10.1534/g3.117.300148 (PMC5677164; doi:10.1534/g3.117.300148)
Supplement: Supplementary file 5 [file 3719TableS3.docx]

Table S3. Genotype counts for progeny from reciprocal backcrosses between IM62 and the doubly heterozygous IL-N (*hms1*_GN_; *hms2*_GN_).

|  |  | *hms1*; *hms2* genotype | | | |
| --- | --- | --- | --- | --- | --- |
| Maternal parent |  | GG; GG | GG; GN | GN; GG | GN; GN |
| IL-N |  | 105 | 45 | 105 | 99 |
| IM62 |  | 35 | 3 | 31 | 35 |
